# A large-scale, multitask, multisensory dataset for climate-aware crop monitoring in the US from 2018–2022

**DOI:** 10.1038/s41597-026-06611-x

**Published:** 2026-01-20

**Authors:** Adrian Höhl, Stella Ofori-Ampofo, Miguel-Ángel Fernández-Torres, Rıdvan Salih Kuzu, Xiao Xiang Zhu

**Affiliations:** 1 Data Science in Earth Observation, Technical University of Munich (TUM), 80333 Munich, Germany; 2 Munich Center for Machine Learning, 80333 Munich, Germany; 3 Department of Signal Theory and Communications, Universidad Carlos III de Madrid (UC3M), 28911 Madrid, Spain; 4 Remote Sensing Institute, German Aerospace Center (DLR), 82234 Wessling, Germany

**Keywords:** Environmental sciences, Agroecology, Ecology

## Abstract

The escalating challenges of climate change, extreme weather events, and increasing food demand impose a significant strain on global food production. To develop and apply sustainable agriculture practices, farmers and organizations require detailed, timely information about weather, crops, and yields. While efficient agricultural monitoring relies heavily on remote sensing, the existing literature suffers from a notable lack of comprehensive, large-scale crop monitoring datasets. This paper introduces CropClimateX, a novel database built by optimizing location sampling to substantially cover cultivated areas throughout the contiguous United States. The database comprises 15,500 small 12 × 12 km data cubes spanning 1,527 counties. Crucially, each data cube integrates a rich array of multi-source information, including multi-sensor imagery (Sentinel-1/2, Landsat-8, MODIS), weather and extreme events (Daymet, heat/cold waves, and drought monitor maps), and environmental features (soil and terrain characteristics). This comprehensive, integrated dataset is designed to support a wide range of agricultural monitoring tasks, providing a vital resource for advancing research in sustainable farming and crop modeling.

## Background & Summary

As global warming progresses, extreme climatic conditions are expected to intensify^1^. These extreme weather events are noticeable in major food-producing regions such as the United States (US). For instance, during the 2012 drought, corn yield decreased by over 20%, subsequently increasing corn price by over 50%^2^. Therefore, climate change, intensified by increasing global population rates and living standards, threatens food production worldwide. This directly impacts one of the major United Nations (UN) Sustainable Development Goals: achieving food security and ending hunger^3^. Monitoring crops is essential to controlling, gaining insights, and developing agricultural practices that foster more resilient and sustainable food production^4^. Crop monitoring supports farmers’ management decisions, such as irrigation and nutrition management, sowing and harvest planning, and hazard preparation. It helps assess crop health, crop yield, and the impact of climate change on crops. Therefore, gaining knowledge about future yields, forecasting and detecting extremes, estimating crop health, stress, and phenology, as well as the prognosis of influences on vegetation and the environment, are vital tasks for achieving food security.

Remote Sensing (RS) plays a pivotal role in agriculture and climate studies and has become a primary method for gathering crop information^5–7^. High-dimensional spatial, spectral, and temporal data resulting from frequent revisits of the Earth are improving our understanding of changes on the Earth’s surface. Existing agriculture works often utilize satellite-based surface reflectance, vegetation indices (VIs), weather, and soil data^8,9^. However, the increasing number of satellites monitoring the Earth generates vast amounts of data, which presents a big challenge for constructing large, multifaceted datasets. Simply collating imagery for entire regions would lead to an unmanageable amount of data. Thus, no benchmark dataset currently integrates these diverse data sources. As such, researchers typically focus on specific areas and sensors, limiting the scope of their studies. A solid and optimized data collection is essential for supporting the development of new methods for processing and analyzing data for agriculture monitoring.

Machine Learning (ML) and Deep Learning (DL) have shown exceptional performance in numerous Earth observation (EO) tasks^10^ and have also emerged as a cornerstone in agricultural monitoring, offering state-of-the-art performance, particularly in processing large-scale remote sensing data. The effectiveness of these models is highly dependent on the availability of curated, labeled, and application-specific datasets. However, comparing methodological advances in research studies remains challenging due to the common use of different sets of various sensors, time frames, crop types, and target variables. This challenge highlights the need for a comprehensive dataset.

Most crop yield datasets^11–15^ are small-scale, often lacking RS data, or they only include image histograms or VIs. Two datasets focus on integrating multiple sensors for crop monitoring. Agriculture-Vision^16^ utilizes aerial images with a resolution of up to 10 cm, making it a small-scale dataset primarily aimed at segmentation tasks. SICKLE^17^ collects a small-scale, multi-task, satellite imagery database in India. Only one large-scale database^18^ addresses crop yield. It covers the US, but relies on a naive solution for generating the minicubes for each county. The authors provide 40 m resolution data and three bands of Sentinel-2, along with weather information.

On the other hand, the majority of the datasets related to extreme events predominantly focus on drought indices at either global^19–21^ or regional scale^22–24^. Others aim to predict meteorological variables and thus, (extreme) weather events^25,26^. The EarthNet initiative^27,28^ seeks to connect this type of data with RS by incorporating and forecasting Sentinel-2 images across Europe. The DeepExtremeCubes dataset^29^ covers compound heat and drought events worldwide, offering Sentinel-2, Digital Elevation Model (DEM), and land cover information, but focuses on non-crop vegetation responses.

Therefore, there is still a need for large-scale databases focused on agricultural regions. Especially, in the era of foundation models^30^. Additionally, existing databases often lack comprehensive features such as climate, weather, extreme event indices, soil, terrain, various RS sensors, and crop management data, and current literature emphasizes the need for multimodal remote sensing data in crop monitoring^31^. To address these gaps, we present CropClimateX, a large-scale dataset comprising 15,500 small data cubes (i.e., minicubes), each with a spatial coverage of 12 × 12 km, spanning 1,527 counties in the contiguous US from 2018 to 2022. This dataset includes satellite imagery with various resolutions/sensors, and additional input features (weather, soil, terrain), designed to support the development of advanced ML and DL models for agricultural monitoring. An overview and comparison with the existing databases is shown in Table 1. By offering a wide range of features, CropClimateX enables a united multi-task setting for various experimental scenarios for multiple crop and climate applications, including crop yield prediction, phenology mapping, crop condition forecasting, extreme weather event detection and prediction, sensor fusion, pretraining on crop areas, and multi-task learning. Our methodological approach for efficient minicube sampling employs a Genetic Algorithm (GA) and a Sliding Grid Algorithm (SGA) to approximate optimal locations for the minicubes, specifically targeting cultivated areas. This strategy reduces the overall dataset size by 43% while retaining 93% of the crop regions.

**Table 1:** Related databases to CropClimateX in the literature, structured by task.

Dataset	Task	Region	Time	Features							Target	Format			
				Surf. Refl.	Veg. Index	Weather	Climate	Soil	Terrain	Mgmt.		Tab./Img.	MultiRS	Opt.	Size
Duden *et al*.^83^	yield	Germany	1979–2021							*✓*	county	T			<100MB
ACDC^11^	yield	USA	1981–2015			*✓*		*✓*			county	T			<100MB
GDHY^14^	yield	Global	2000–2016		*✓*	*✓*					pixel	I			<100MB
GlobalWheatYield4km^15^	yield	Global	1982–2020		*✓*	*✓*		*✓*			pixel	I			<10GB
CY-Bench^84^	yield	Global	2003–2024		*✓*	*✓*		*✓*		*✓*	county/district	T			<10GB
CropNet^18^	yield	USA	2017–2022	*✓*	*✓*	*✓*				*✓*	county	I			2.3TB
EarthNet^27,28^	ESF	Europe	2017–2022	*✓*	*✓*	*✓*			✓		pixel	I			600GB
DeepExtremeCubes^29^	ESF (non-crop)	Global	2016–2022	*✓*	*✓*	*✓*	*✓*		*✓*		pixel	I		*✓*	1.8TB
DroughtED^85^	drought	USA	2000–2020			*✓*		*✓*			county	T			<1MB
SustainBench^13^	multi-task	USA Brazil Argentina	2005–2017	*✓*		*✓*					county/pixel	T/I			150GB
SICKLE^17^	multi-task	Tamil Nadu, India	2018–2020	*✓*	*✓*					*✓*	county/pixel	I	*✓*		—
**CropClimateX**	multi-task	USA	2018–2022	*✓*	*✓*	*✓*	*✓*	*✓*	*✓*	*✓*	county/pixel	T+I	*✓*	*✓*	6.3TB

The tasks are crop yield prediction (yield), Earth Surface Forecasting (ESF), drought prediction (drought), or multi-task, when the database aims for more than one task. The geographical and temporal coverage of each dataset is shown in the region and time columns, respectively. The target column specifies the prediction unit of the task (county, wdistrict, pixel), while the format shows how the data is delivered. The database can contain tabular (T) or image (I) data, have multiple sensors for the same region (MultiRS), and be based on an optimized minicube sampling procedure (Opt.). The following abbreviated terms are defined as surf. refl. - surface reflectance, veg. - vegetation, mgmt. - farm management.

## Methods

**Fig. 1:**
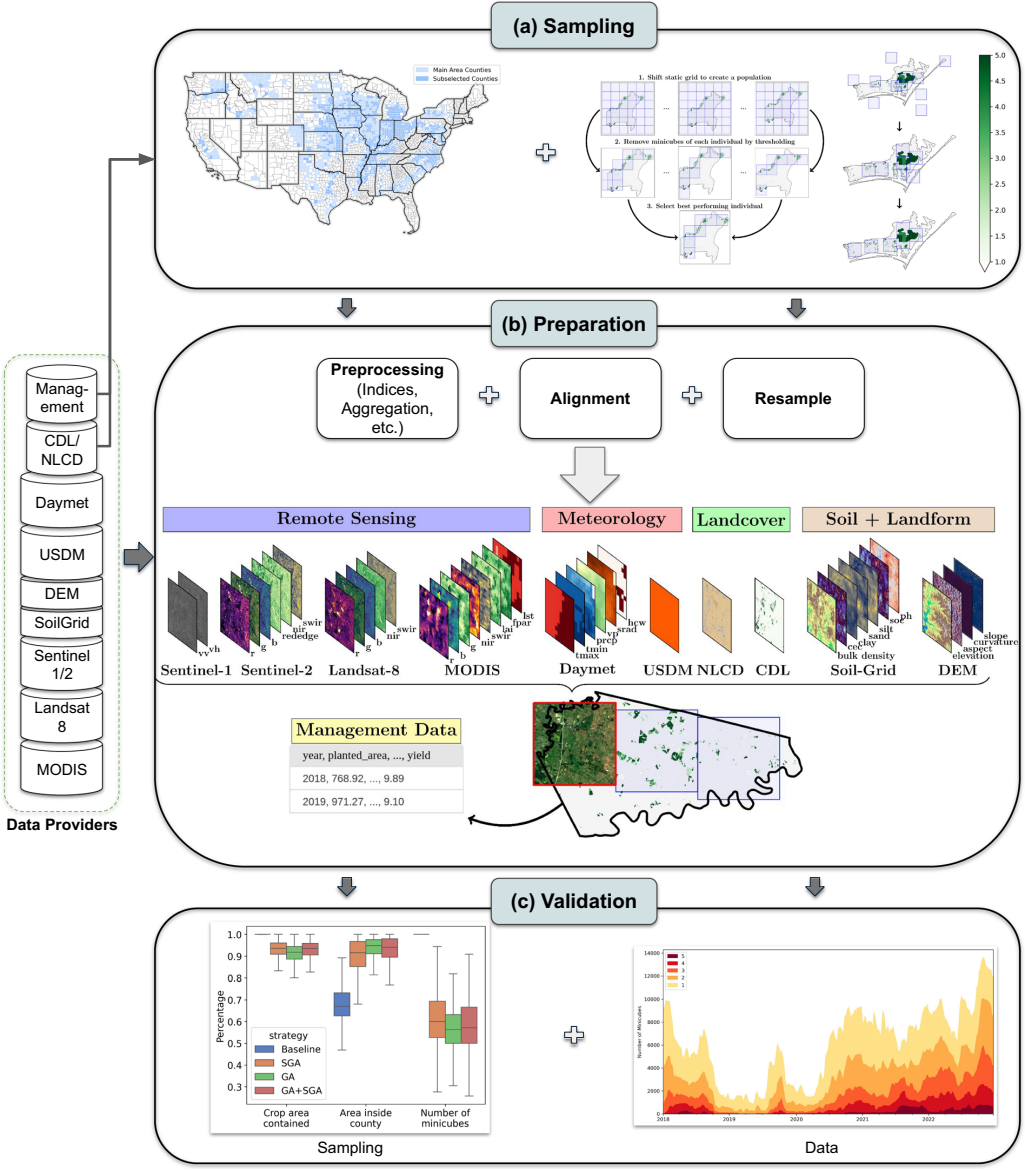
A schematic diagram illustrating the development workflow of CropClimateX. It starts with (a) Sampling: Selection of the counties in the US and optimizing the locations of the minicubes for each county. An enlarged version of these figures is shown in Figs. 2, 3. Data preparation through downloading and preprocessing diverse sources, illustrated by a visualization of the data available for each minicube in the CropClimateX database for an example county (ID: 21239). (c) Validation of the minicube locations and data. An enlarged version of these figures is shown in Figs. 8, 6.

CropClimateX incorporates comprehensive data sources, prepared in data cubes spanning the continental US and covering the years 2018 to 2022. First, counties are sampled to ensure crop and geographic coverage. Second, the locations of the data cubes within each county are spatially optimized to cover agricultural areas. Then, the dataset is built and validated. The complete schematic of our approach can be found in Fig. 1.

### Study Area

The contiguous USA features several climate zones. According to the Köppen climate classification^32^, the southwestern region experiences warm to hot desert and semi-arid climates, while a cold semi-arid climate characterizes other parts of the western USA. A humid subtropical climate is found in the southeastern areas, and the Northern Plains and Midwest experience a humid continental climate. Crop production occurs across these diverse climates. The 2022 agricultural census reported about 2 million farms covering 39% of the total land area with an average size of 463 acres^33^. Corn and soybeans are primary crops largely cultivated in the Midwestern states, while wheat production is concentrated in the Northern Plains, following a north-south distribution. The combination of extensive crop statistics, diverse agro-climatic conditions, and the influence of climate change and variability on agricultural systems^34^ underscores the significance of studying climate-crop interactions in the United States. Furthermore, the projected impacts of extreme weather and climate-related events on economic growth, water availability, and food systems highlight the region’s importance for research on agricultural resilience and climate adaptation^35^.

### Input Data

This section presents the data and processing steps to generate the dataset. Daymet weather data was downloaded from the official website. Satellite and terrain data were sourced from various platforms due to data size and throughput restrictions. Specifically, the following sources were used: Planetary Computer^36^, Google Earth Engine^37^, Copernicus Data Space Ecosystem^38^ (all accessed via Terragon^39^), and Sentinel Hub.

#### Crop and Land Management

The National Agriculture Statistic Service (NASS) of the United States Department of Agriculture (USDA) conducts yearly surveys on agricultural production and economics. The data is openly accessible through the QuickStats database^40^. We retrieved annual *crop yield* at the county level, selecting only those corresponding to our target counties, crops, and years. In addition to the yield records, we incorporate county-level farm management information, including area planted, area harvested, and production quantities. We convert yield statistics to tonnes per hectare. Planted and harvested areas are provided in hectares, whereas production estimates are in tonnes. Although NASS has separate yield estimates for irrigated and non-irrigated farms, the crop type maps do not decipher these systems. Therefore, we used the undifferentiated yield record.

Annual *crop type* maps are produced by USDA National Agricultural Statistics Service, Research and Development Division, Geospatial Information Branch, Spatial Analysis Research Section^41^. The 30 m maps are a CONUS-wide product of ML analysis that integrates moderate-resolution satellite imagery with extensive agricultural ground reference. The maps contain agricultural land use and other land cover types. The USDA^42^ provides the usual planting and harvest periods.

The National *Land Cover* Database (NLCD)^43^ is produced by the Multi-Resolution Land Characteristics (MRLC) Consortium, a partnership of federal agencies led by the US Geological Survey. The archive contains 30 m land cover maps for the conterminous USA at a 2–3 year interval since 2001. Over 20 classes, including open water, agricultural land use, and built environments, are represented in each product.

#### Satellite Data

We include the most commonly used satellites in agriculture, namely Sentinel-2, Landsat-8, and MODIS. These satellites provide multiple short-wave infrared (SWIR) bands and, in the case of Sentinel-2, also red edge bands. These spectra are reported to be valuable for agricultural and vegetation tasks for Sentinel-2^44–49^ as well as Landsat-8^45,47^. Following previous works^44,50^ that demonstrated a high correlation among these bands, we consider only one band of each spectral group to keep the data size manageable. Furthermore, the Sentinel-2 red edge band B8A band is not included since the near-infrared (NIR) encompasses it.

First, *Sentinel-2* consists of two identical platforms with the same multispectral instrument, providing a spatial resolution of 10 m to 60 m for 13 spectral bands and a combined temporal resolution of five days. We resample the L2A product^51^ to the coarsest resolution of the selected bands, 20 m, and select the least cloud-occupied image within a 15-day window. Second, the *Landsat-8* satellite features a multispectral instrument with a 30 m spatial resolution for visible, NIR, and SWIR bands, operating at a 16-day temporal resolution^52^. To simplify data handling, the dates of the Landsat-8 and Sentinel-2 time steps have been aligned to start from the first day of every year. For example, Landsat-8 has a temporal resolution of 16 days, so images dated January 1, 2022, were captured between January 1, 2022, and January 16, 2022. Third, the *Moderate Resolution Imaging Spectroradiometer (MODIS)* MOD09A1-061^53^ provides an estimated surface reflectance across seven spectral wavelengths. The atmospherically corrected reflectance ranges from visible to SWIR regions. The bands have a spatial resolution of 500 m and a temporal resolution of 8 days. For each pixel, a value is selected from all acquisitions within the 8-day composite period, considering the criteria: low view angle, absence of aerosol lading, cloud, and cloud shadow. In addition to the surface reflectance data, we include the *MODIS Leaf-Area Index (LAI) and Fraction of absorbed Photosynthetic Active Radiation (FAPAR)* 15A2H-061 product^54^, which has the same resolution, and the *MODIS land surface temperature (LST)* 11A2-061 product^55^ with 1 km spatial and an 8-day temporal resolution. LAI and FAPAR provide critical biophysical information that complements surface reflectance data to understand plant health and productivity.

Additionally, we include radar data from *Sentinel-1*^56^. In our time and area of interest, the Sentinel-1 consists of two platforms with 10 m resolution, two polarizations, and ascending and descending orbits. The combined temporal resolution is 6 days. However, it fluctuates heavily over the USA, and sometimes only one orbit direction is available. Also, one platform was discontinued in 2022. Hence, we resample it to a 12-day resolution for both ascending and descending orbits separately, while removing platforms that have fewer than 5 time steps in a year. Further, we resample to 20m spatial resolution to align with Sentinel-2. We choose the Radiometrically Terrain Corrected (RTC) product, which further processes the Ground Range Detected (GRD) data to correct geometric distortions, normalizes the backscatter values radiometrically, and orthorectifies the data.

*Vegetation indices:* Understanding spectral characteristics in relation to the Earth’s surface facilitates the development of indices that highlight specific spectral reflections, such as vegetation greenness, stress, or water content. Our band selection allows the computation of commonly used spectral indices, such as the Normalized Difference Vegetation Index (NDVI), the Enhanced Vegetation Index (EVI), or the Normalized Difference Water Index (NDWI), to support crop monitoring^57,58^. These indices can be easily derived from the available data; therefore, they have not been preprocessed to conserve storage.

#### Weather and Climate

*Daymet* delivers continuous daily surface weather and climatological summaries at 1 km over continental North America^59^. The estimated parameters, including minimum and maximum temperature, precipitation, shortwave radiation, vapor pressure, and snow-water equivalent, are derived from meteorological station data and various supporting sources using statistical modeling techniques. The data is created by the Environment Sciences Division of Oak Ridge National Laboratory.

Our derived Heat and Cold Wave (HCW) index follows the implementation of the European Drought Observatory (EDO) using Daymet temperature variables. The EDO applies HCW for detecting and characterizing periods of extreme temperature anomalies (heat and cold waves) in Europe. The index is derived from daily minimum and maximum temperatures based on the persistence for at least three consecutive days of events, where the daily threshold of minimum and maximum temperatures are beyond or below the *x*^*th*^ percentiles^60^. Our index includes the 1^*th*^, 5^*th*^, 10^*th*^, 90^*th*^, 95^*th*^, and 99^*th*^ percentiles derived from 1980 to 2022.

*The US Drought Monitor (USDM)*^61^ is a national repository to monitor the frequency, severity, and duration of droughts in the United States. The drought index is produced jointly by the National Drought Mitigation Center (NDMC), the University of Nebraska-Lincoln, the National Oceanic and Atmospheric Administration (NOAA), and the USDA. It employs multi-source inputs such as the Palmer Drought Severity Index (PDSI), the Standardized Precipitation Index (SPI), and satellite-based vegetation health and soil moisture indicators, leveraging local expert knowledge via a network of observers, which includes climatologists and hydrologists, to create five drought stages. The drought classes are encoded as integers from zero to five: zero being normal or wet conditions, while drought categories are defined from abnormally dry (D0) to exceptional drought conditions (D4).

#### Soil Properties

As a key resource for global digital soil mapping, *SoilGrid*^62^ leverages geo-referenced soil profile data from the World Soil Information Service (WoSIS), which integrates contributions from various national and international agencies. In the case of the United States, a significant portion of the soil data is sourced from the National Cooperative Soil Survey (NCSS) and the National Cooperative Soil Characterization Database. Predictions of several soil properties are available at varying soil depths and a spatial resolution of 250 m. Soil properties are essential for crop productivity due to their direct influence on soil fertility, water retention, and nutrient availability^63,64^. They have been widely used in crop yield studies^65^ and have been shown to enhance yield prediction accuracy^66,67^. For each soil property, the values were averaged at depths 0 to 100 cm to generate a single representative value for the entire depth profile. Although crops such as maize and wheat can have deeper roots, significant water, and nutrient uptake for most crops is typical at the chosen depth^68,69^.

#### Terrain Features

Terrain information, such as elevation, offers valuable insight into the variability within agricultural fields, which affects drainage patterns and soil composition. USGS provides a *DEM* from the 3D Elevation Program (3DEP)^70^. The data is available across the USA and its territories at several spatial resolutions. For this study, we retrieved the 30 m product over the conterminous USA. In addition, we derived slope, aspect, and curvature from the elevation model. All terrain-related features are available as static features in our database.

### Regional Sampling Strategy

**Fig. 2:**
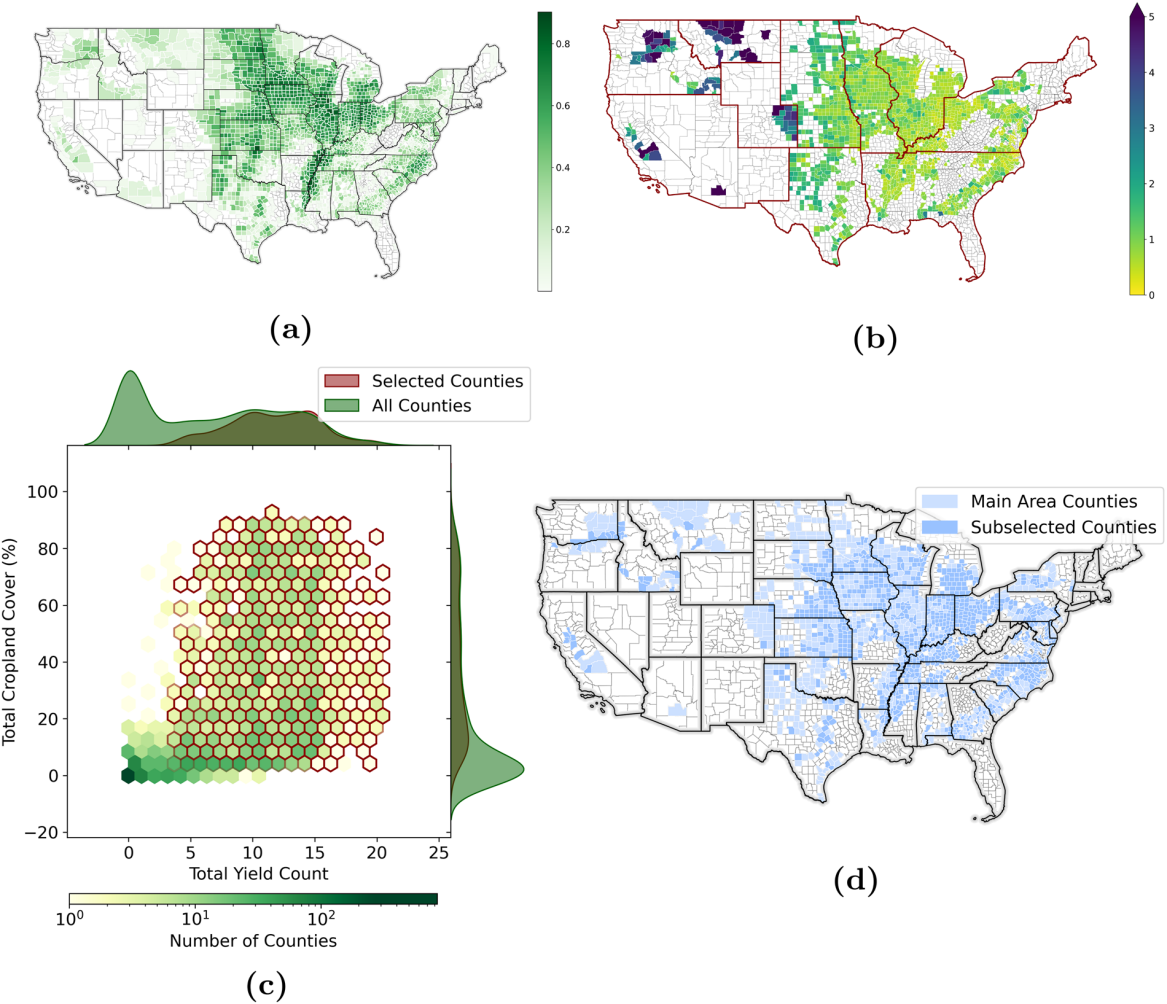
Validation of the county sampling. (**a**) The yield report and crop coverage composite score (higher is better) (**b**) The ratio of number of minicubes with the yield reports of the main study area (smaller is better, the red edges separate different clusters) (**c**) The area covered by cropland and the reported yield for all of the counties in the continental US (green) and the selected counties for this study (red) are shown in a 2D histogram (center), and the distributions for both variables (x and y axes). The highest frequencies are found in counties with a low number of crops and a low yield report count. We selected only counties with a high amount of crop areas as well as high reporting numbers (dark red). (**d**) Selected counties for the CropClimateX dataset (light blue presents the main study area and dark blue the sub-selection for Sentinel).

To develop an effective agriculture monitoring dataset in the US, crop types and counties were filtered based on reported yield frequency and spatial coverage from 2018 to 2022, allowing for a precise representation of cropland regions while excluding other non-agricultural areas but, at the same time, resulting in a sufficient number of unique target crop yield values to train supervised ML models for this task. According to the USDA crop survey database, the five most reported crops were chosen, i.e., corn, soybean, winter wheat, cotton, and oats. Also, these crop types provide comprehensive geographic coverage of the US. Each county must have a spatial crop coverage of more than 5% and, at least, three years of yield records. Additionally, a more effective county selection is achieved by defining a ratio of target crop yield values to data cubes. This ratio assesses the data size needed to represent a county and the potential yield frequency. It helps to identify and discard counties with a high number of data cubes but low yield reporting frequency. This selection process results in a total of 1,527 counties and forms our main study area (see Fig. 2d in light and dark blue color). Furthermore, to ensure a manageable data size for the higher resolution Sentinel-2 sensor, a subset is defined, removing additional counties with a low ratio value. To keep the spatial representation, the counties are grouped into spatial clusters (see Fig. 2b) and regions with fewer counties are preserved, even if this ratio is more favorable in others. The distribution of counties for this sub-selection is given in Fig. 2d in dark blue and results in 911 counties.

### Minicube Sampling Strategy

The annotations in this dataset are pixel- or county-based. However, the size of the counties varies greatly from 150 to 50,000 km^2^. Hence, representing the input county-wise for training Deep Neural Networks (DNNs) is not feasible. To overcome this, the spatiotemporal data can be organized in smaller data cubes, each covering a subset of the extent, also known as minicubes. These minicubes have a spatiotemporal grid, an intuitive and well-structured format, consistent with the concept of the larger Earth System Data Cubes^71^. Consequently, the data for a county is represented by a variable number of minicubes, enabling modular and efficient storage and targeted processing.

One minicube encompasses 12 × 12 km, which contains multiple agricultural fields and management zones. This size maintains internal spatial coherence at resolutions of Sentinel-1/2 (20 m) and Landsat-8 (30 m), MODIS (500 m), and Daymet (1000 m), resulting in pixel side lengths of 600, 400, 24, and 12 pixels, respectively, which are sufficient for training DL models without excessive computational overhead. Comparable spatial partitions have been employed for regional-scale yield modeling in CropNet^18^, with an edge size of 9 km, while other crop datasets do not use minicubes to represent the data. In contrast, other minicube datasets, such as DeepExtremeCubes^29^ and EarthNet2021^27^, use a smaller edge size of around 2.5 km, which can also be achieved by splitting one minicube into smaller regions.

A large-scale dataset covering the USA quickly exceeds a manageable size. Further, in agricultural monitoring, regions other than agriculture, like large cities or water bodies, do not need to be represented on a large scale. Hence, it is impractical to cover all counties entirely. To reduce the size of the dataset, we select minicubes where crops grow.

We aim to select each county’s agricultural regions so that minicubes cover the main areas, approximating the crop areas and yields. At the same time, the minicubes should not overlap with each other nor exceed the boundary of a county. Additionally, all minicubes should have the same height and width without varying in size. Trying all combinations of minicubes brute-force to cover the area of a county would be infeasible. To the best of our knowledge, no exact solution exists for this problem within a reasonable amount of time, and similar packaging or coverage problems are known to be NP-hard^72^. Many approaches have been proposed to approximate solutions, but all are specialized in distinctive tasks. Location Set Covering Problems (LSCPs) have been formulated by relying on point or area demands for facility coverage^73,74^, but this is still computationally expensive. Also, a few strategies have been proposed to optimize Unmanned Aerial Vehicle (UAV) paths, including numerical optimization methods, genetic algorithms, and particle swarm optimization^75,76^.

Relying on the works above, we followed two similar strategies to approximate a solution for minicube sampling. The two strategies are compared to the straightforward solution of overlaying a grid and removing minicubes whose area is outside the county. This constitutes our baseline and was already followed by CropNet^18^.

The algorithms consider four variables to define and optimize a heuristic: (1) the proportion of the area of the minicubes within the county to the total area of the minicubes, (2) the overlap between the minicubes with respect to the total area of minicubes, (3) the number of minicubes with respect to the number of minicubes in the baseline, and (4) the crop area contained by the minicubes with respect to the crop area contained by the county. These variables are aggregated through a weighted sum that defines the quality of each individual. The weights were empirically determined by tuning them on a representative subset of counties and retaining the configuration that produced the best overall performance. The reader is referred to the Supplementary Information for further details.

**Fig. 3:**
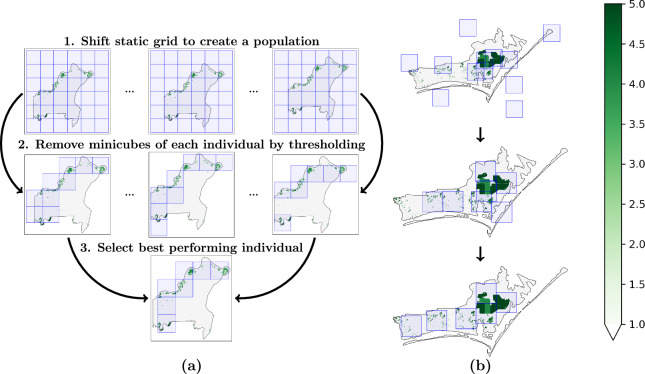
(**a**) Visualization for the three steps of the Sliding Grid Algorithm (SGA) and an example county (ID: 29189). (**b**) Sample steps from the first generation, generation 15, and the last generation for the Genetic Algorithm (GA) and an example county (ID: 37031). The accumulated number of crops is in green, the sampled minicubes are in blue, and the county outline is in black.

As a first approach, we introduce the Sliding Grid Algorithm (SGA) (see Algorithm 1), overlaying a grid over the county and sliding that grid pixel-wise over the area until one minicube size is shifted. Each shift constitutes one individual of a population. Next, the minicubes of each individual are removed by thresholding the area outside of the county and the amount of crop within a minicube. Ultimately, the best-performing individual, according to the score introduced above, is selected. The three steps of the algorithm are illustrated in Fig. 3a. Relying on a fixed grid structure instead of unrestricted positions significantly reduces the algorithm’s runtime. Besides, we scale the number of shifts linearly by the size of the county.

#### Algorithm 1

Sliding Grid Algorithm (SGA).

Our second approach (see Algorithm 2) leverages a Genetic Algorithm (GA) to optimize minicube sampling. We use the Python package DEAP^77^ for our implementation. The score introduced above defines the individual’s fitness. The algorithm begins with a randomly initialized population, where each individual contains random minicube positions, referred to as the first generation. Each population then creates a new generation by producing offspring through the application of mutation, crossover, and selection. All operations are applied to randomly selected individuals of the current generation. The mutation shifts a minicube or adds/removes a minicube from an individual. By applying crossover, minicubes are exchanged between individuals. In the end, the best offspring is selected using a roulette strategy, where the best individual is iteratively selected from a pool of randomly chosen individuals. These steps are repeated until the result does not improve or the maximum number of generations is reached. The best individuals from three generations and an example county are visualized in Fig. 3b as an example. To account for randomness and the potential chance of achieving a bad solution, the algorithm is run three times on each county, and the execution achieving the best fitness is chosen.

#### Algorithm 2

Genetic Algorithm (GA).

Ultimately, the final locations of the minicubes are determined by combining the best results from both approaches, consistently selecting minicubes from the most successful approach at the county level.

## Data Records

The CropClimateX^78^ dataset is available for unlimited use under the Creative Commons License 4.0 International. It can be found at Huggingface (https://huggingface.co/datasets/torchgeo/CropClimateX) in the ZARR format^79^, allowing compression, grouping, and automatically applying offsets and scaling when loading with Xarray^80^. Xarray is suggested for reading the files to utilize the full functionality. We recommend having more than 6.3TB of free space to download the entire database. However, the 1527 counties and features can be downloaded individually, allowing for more flexibility in managing the size. The data can be downloaded from the website or via API. A script for downloading is also available in the implementation. An overview of the dataset variables and their size is provided in Table 2.

**Table 2:** Data sources covered by the CropClimateX dataset.

Data source	Band Descriptions	Number of bands	Time	Spatial Resolution (m)	Temporal Resolution	Total Size
Sentinel-1	VV, VH	2	2018–2022	20	6 days	1.3 TB
Sentinel-2	RGB, NIR, SWIR, Red-Edge	6	2018–2022	20	15 days*	2.5 TB
Landsat-8	RGB, NIR, SWIR	5	2018–2022	30	16 days	2.1 TB
MODIS	RGB, NIR, SWIR, LAI, FPAR, LST	9	2018–2022	500/1000	8 days	56 GB
Daymet	Temperature, Precipitation, Vapor Pressure, Shortwave Radiation, Climate for the former variables from 1980-2017, Heat/Cold-wave Index	6	2018–2022	1000	1 day	225 GB
USDM	US Drought Monitor	1	2018–2022	1000	7 days	18 GB
CDL	Crop Cover	1	2018–2022	30	1 year	4.6 GB
NLCD	Land Cover	1	2018–2022	30	2–3 years	2.2 GB
Management data	Yield, Production, Harvested & Planted Area	4	2018–2022	—	1 year	1.6 MB
DEM	Elevation, Slope, Aspect, Curvature	4	static	30	static	30 GB
Soil	Bulk Density, Cation Exchange Capacity, Organic Carbon Concentration, pH, Proportions of Clay/Sand/Silt	7	static	30	static	3.2 GB

A cloud mask layer accompanies each multispectral satellite source. Rows marked with * indicate data resampled using the least cloud-occupied image available.

## Technical Validation

### Input Data

**Fig. 4:**
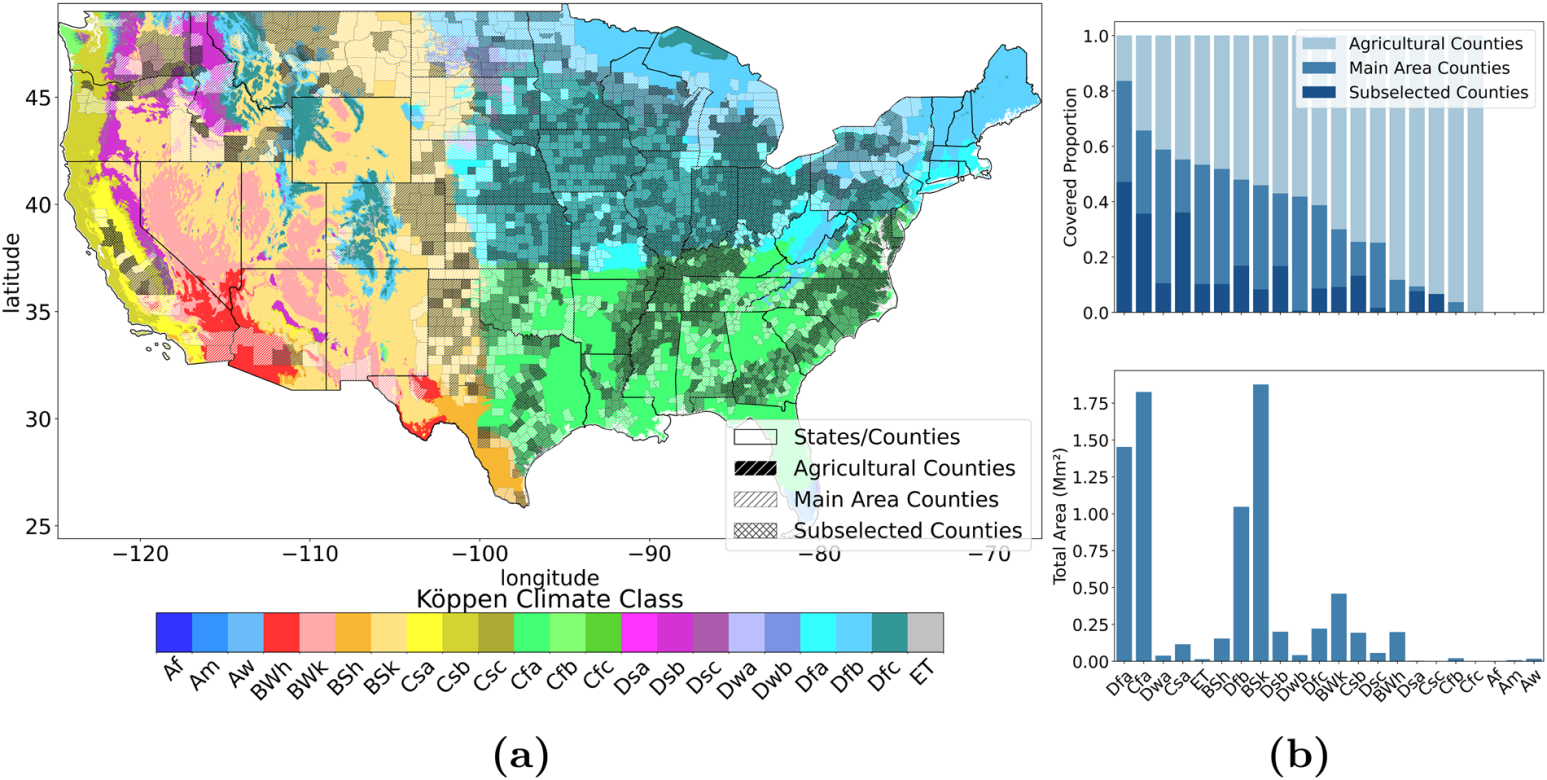
A visualization of the Köppen climate regions in the continental US. (**a**) Spatial visualization: agricultural counties (with more than 1% cropland and reporting more than one yield value) are shaded white; included counties are shaded black, and the subset is cross-shaded black. (**b**) The coverage of the different climate zones as a proportion of their size and the total area covered in the continental US by each climate zone. The climate zone acronyms are: Af - Tropical, rainforest; Am - Tropical, monsoon; Aw - Tropical, savannah; BWh - Arid, desert, hot; BWk - Arid, desert, cold; BSh - Arid, steppe, hot; BSk - Arid, steppe, cold; Csa - Temperate, dry summer, hot summer; Csb - Temperate, dry summer, warm summer; Csc - Temperate, dry summer, cold summer; Cfa - Temperate, no dry season, hot summer; Cfb - Temperate, no dry season, warm; Cfc - Temperate, no dry season, cold; Dsa - Cold, dry summer, hot summer; Dsb - Cold, dry summer, warm summer; Dsc - Cold, dry summer, cold summer; Dwa - Cold, dry winter, hot summer; Dwb - Cold, dry winter, warm summer; Dfa - Cold, no dry season, hot summer; Dfb - Cold, no dry season, warm summer; Dfc - Cold, no dry season, cold summer; ET - Polar, tundra.

According to the Köppen climate classification^81^, there are 22 climate zones in the continental US. However, crop land does not cover all climate zones equally. Counties reporting yield and having more than 1% crop area (white - referenced as Agricultural Counties) are shown in Fig. 4a. As can be seen, the north-eastern (US Corn Belt) region is heavily utilized for crop production, whereas the southern and western regions, with different climates, have less cropland for the crops included in this dataset. The total area of the climate regions in the US (lower plot) and the covered proportions by the agricultural counties and our selection are then shown in Fig. 4b. While there is good coverage of the four major climate zones (Dfa, Cfa, Dfb, BSk), smaller areas are not covered as well as bigger areas, e.g., the temperate - no dry season - cold (Cfc) zone only covers around 260km^2^ and is not included in the dataset. Furthermore, the subselection has different coverage than the main area and does not include some regions that are part of the main area (Dwb). Still, most climate zones are considered, offering a good estimate of the diversity of the climate zones in the US.

**Fig. 5:**
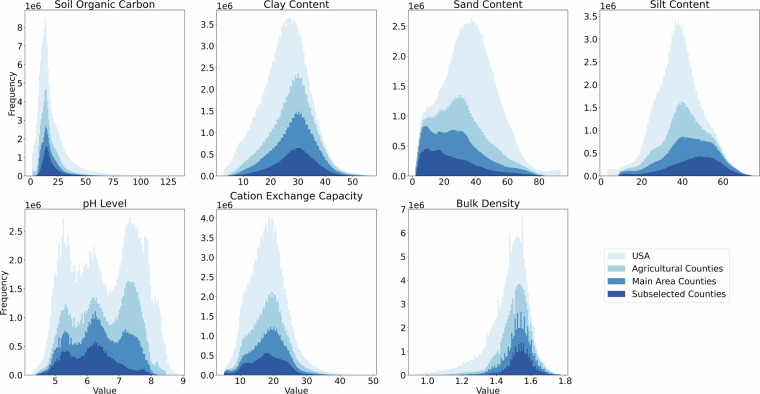
Distribution of soil parameters in the US, agricultural areas (counties with more than 1% cropland and reporting more than one yield value), and the selected counties in the CropClimateX dataset.

Figure 5 shows the distribution of soil properties. The overall distribution differs significantly from the agricultural distribution, for example, in terms of sand and silt content, because the amount of soil parameters is geographically not uniformly distributed across the US, e.g., the US Corn Belt has a lower amount of sand, while Florida and western parts, like California and Oregon, have a higher amount of sand. Our selection follows the distribution of the agricultural regions in the US. However, there are small deviations for the variables sand/silt content and for the subselection for the pH level. Nevertheless, our selection does not ignore any data ranges.

**Fig. 6:**
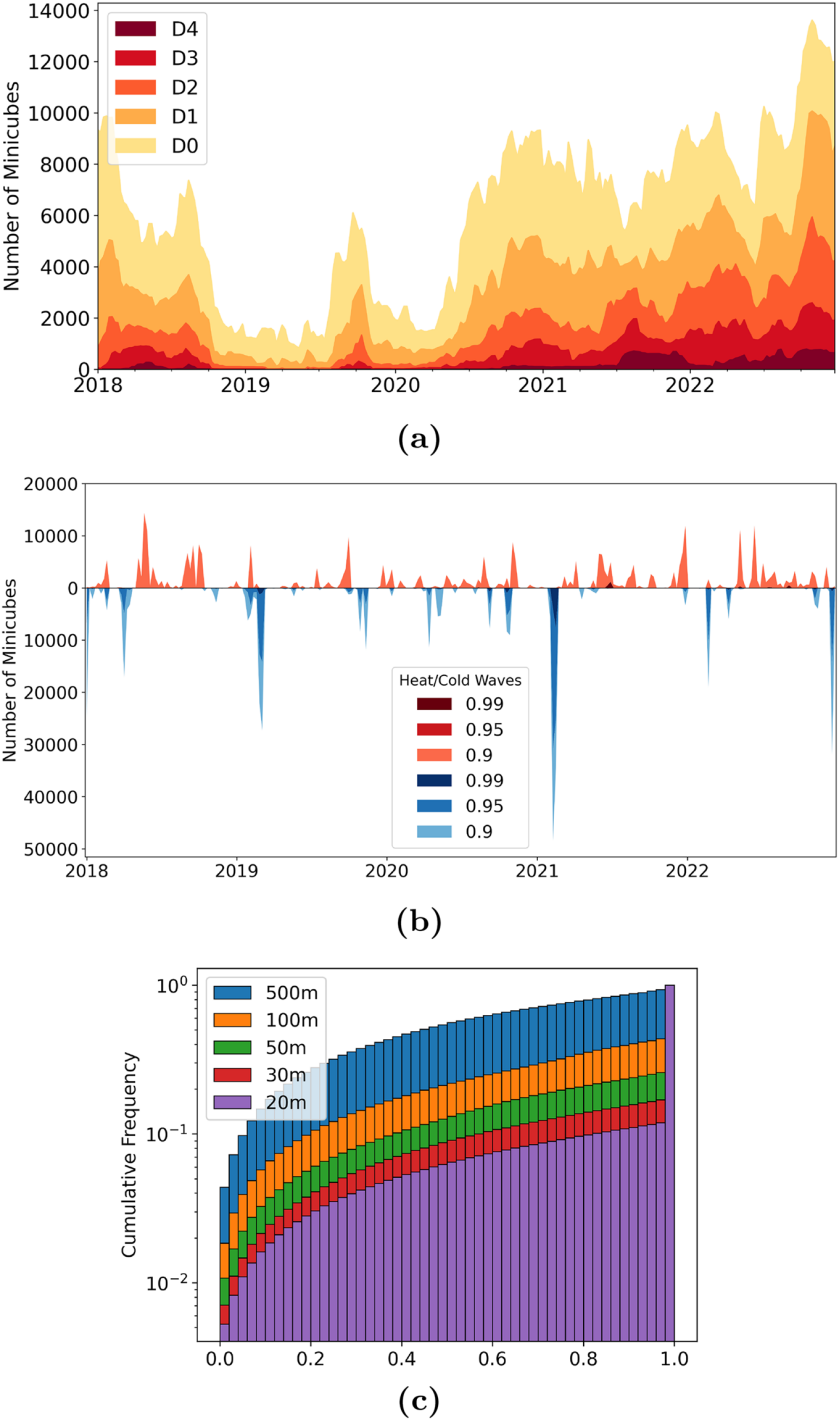
Validation of the data sources. (**a**) Number of minicubes affected by drought conditions of the US Drought Monitor (USDM) (the legend refers to D0: Abnormally Dry, D1: Moderate Drought, D2: Severe Drought, D3: Extreme Drought, and D4: Exceptional Drought). (**b**) Number of minicubes affected by Heat and Cold Waves (HCW) index binned to weekly resolution for visualization purposes (the legend indicates heat and cold 0.9, 0.95, and 0.99 percentiles). (**c**) Logarithmic cumulative frequency of the pixel purity of 100 randomly selected minicubes in the year 2018 for different spatial resolutions (MODIS - 500 m, Landsat-8-30 m, Sentinel-2-20 m).

Moderate Drought (D1) conditions, as reported by the U.S. Drought Monitor (USDM), affected 99% of the minicubes at least once over the past five years. Among those, 76% experienced D2 conditions, 44% faced D3 conditions, and 19% were classified at least once as experiencing the highest level of drought, Exceptional Drought (D4). While 2022 and 2021 were notably drier years, 2019 and 2020 were relatively wet. Heat and cold waves, as described in the HCW index, are typically local phenomena that occur over a short period. While heat waves generally occur in the summer and cold waves in the winter, there were notable exceptions in 2018, when a cold wave struck during spring, and in 2020, when the summer was unusually cold for a short period of time. All minicubes were impacted by these extreme temperature conditions, but only 25% were categorized in the highest percentile for cold waves, while just 4.5% are in the highest percentile for heat waves. An overview of the indices is presented in Fig. 6a,b.

In this analysis, we also focus on evaluating the purity of agricultural pixels for the selected crop types by examining 100 randomly selected minicubes from the year 2018 in Iowa. This assessment utilizes the USDA field boundary product^82^, which is derived from the crop data layer and aerial images from the National Agriculture Imagery Program (NAIP). The Sentinel-2 and Landsat-8 satellites provide higher-resolution imagery compared to MODIS. As a result, they generally achieve significantly higher purity values. We found that, on average, MODIS achieved a purity of 47.8%. In contrast, the Sentinel-2 and Landsat-8 imagery have a higher purity rate, denoted as 93.7% and 91.2%, respectively. This indicates the clear advantage that higher-resolution satellite data offers in terms of accurately distinguishing between agricultural regions and crop types. The relationship between satellite resolution and crop pixel purity is further illustrated in Fig. 6c. This figure visually represents the logarithmic cumulative pixel purity across all assessed crop classes for different spatial resolutions. The trends depicted clearly showcase the benefits of utilizing higher-resolution data for agricultural monitoring, such as Sentinel-2 or Landsat-8, as a significant increase in purity associated with fine-resolution images can be observed. Consequently, these findings underscore the importance of resolution in RS applications aimed at agricultural analysis and monitoring, as they reduce interference from background signals such as other crop types, non-crop vegetation, water bodies, and man-made structures, which can obscure the crop signal.

### County and Minicube Sampling

**Fig. 7:**
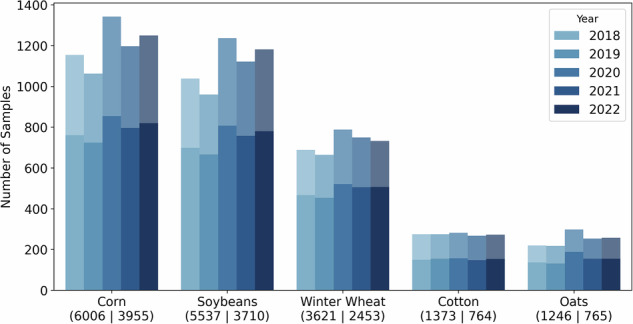
The number of crop labels in the CropClimateX dataset, grouped by crop type and ordered by year. Bars with higher intensity display the subset selection for Sentinel, while bars with lower intensity display the entire dataset. The numbers below the crop type represent the total number of labels, listed in order (main area ∣ subset).

Performance details and a comparison between the SGA and GA approaches are presented in this section. Additional information on the algorithm optimization can be found in the Minicube Optimization section of the Supplementary Information. We first select counties based on the area of crop grown and the yield labels reported. Figure 2c shows the interaction of these two variables. A hexagonal histogram is shown in the center, containing all the counties of the US. The bins with an edge color are selected for our dataset. The highest frequency appears where the lowest amount of crop is grown and where the lowest reporting rate is. As can be seen in the distribution shown on the margins, only the counties with a high number of crop area and yield labels are selected. In summary, our dataset provides the most closely monitored agricultural regions in the US (see the spatial overview in Fig. 2a). It should also be noted that we consider a sub-selection of counties for Sentinel data, which favors counties with a low number of minicubes to reduce the data size. The counties are grouped into clusters to keep a geographic representation. Then, the counties with the lowest ratios of target crop yield values to minicubes are removed from each cluster, as described in the Methods - Regional Sampling Strategy section. A visualization of the ratio of the number of minicubes and yield reports, along with the geographic clusters (red) for the decision can be found in Fig. 2b. This sub-selection allows us to reduce the number of minicubes by over 50% from 15,500 to 7,259 minicubes, while retaining over 911 of 1,527 counties and therefore over 65% of the yield labels. The selected counties and the total number of yield labels for both selections are shown in Fig. 2d and Fig. 7, respectively.

**Fig. 8:**
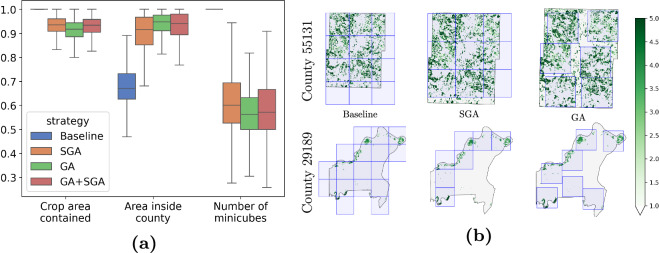
Validation of the minicube sampling. (**a**) Results for the different minicube generation strategies per county. While both Sliding Grid Algorithm (SGA) and Genetic Algorithm (GA) have their strengths, their combination provides a more balanced solution over the different metrics. The number of minicubes was normalized between 0 and 1, considering the maximum number of minicubes for each county (i.e., the baseline always has the highest number of minicubes). (**b**) Minicubes obtained for two example counties (IDs 29189 and 55131) given the baseline, the SGA, and the GA. The accumulated number of crops is in green, the sampled minicubes are in blue, and the county outline is in black.

Figure 8a presents the results of the naive strategy (baseline)^18^, SGA, GA, and the combination of the latter two, for the metrics of crop area covered by the minicubes and county allocation by the minicubes, i.e., the area of the minicubes inside the county. Both strategies outperform the baseline. The SGA and the GA can reduce the number of minicubes from 27202 to 15994 and 14947, which implies a reduction of 41% and 45%, respectively. At the same time, they can cover more than 90% of the crop in most cases. While the baseline has the worst allocation of the counties, with the largest area outside the county, the other strategies can significantly reduce it. Further, we identified two distinct cases of counties, visualized in Fig. 8b together with the corresponding solutions of the algorithms. While one county has a complex shape and agriculture is concentrated in a few areas, the other is nearly rectangular, and the agricultural regions are more uniformly distributed. Both SGA and GA have advantages and disadvantages. On the one hand, GA performs very well in the first case, finding suitable solutions for clustered distributions, while the grid structure limits SGA and cannot place the minicubes more fine-grained in space. On the other hand, GA has issues with the second case: there are more potentially reasonable performing solutions, and it is hard to evaluate them without getting stuck in a local minimum. In this case, SGA has the upper hand because the grid does not allow for breaking its structure, limiting the search space. Additionally, SGA does not provide overlapping minicubes by design, being advantageous in these situations.

Hence, combining the best solutions for each county from SGA and GA allows us to improve the overall results. Their combination covers more agricultural areas, but still reduces the number of minicubes from 27,202 to 15,500. In total, the number of minicubes is reduced by 43%, decreasing the dataset size by the same amount. At the same time, on average, 93% of the crop areas are included.

## Usage Notes

The primary goal of this dataset is to support downstream applications in benchmarking ML and DL models against crop monitoring tasks. It supports a variety of use cases, including crop yield prediction, crop phenology mapping, crop condition forecasting, extreme weather event detection and prediction, sensor fusion, sensor transfer learning, sensor performance comparison, pretraining on crop areas, and multi-task learning. The original sensor resolution was retained to allow full flexibility for the downstream applications. Depending on the task to be carried out, they may need to be harmonized.

The sampling approach does not follow a regular grid, which can limit analyses that rely on regular grids or local heterogeneity, such as nationwide phenology estimation. The dataset only considers a time period from 2018 to 2022 and is biased towards agricultural regions for the selected crop types. Additionally, crop types and extremes are imbalanced across the US. Hence, other crop types or certain weather events may be underrepresented and depend on the geographical data split.

To avoid overestimating model performance, the autocorrelation between neighboring counties and minicubes should be taken into account. Additionally, there can be slight overlaps between minicubes. Depending on the application, the end-user should make sure they do not end up in different data splits.

## Supplementary information

Supplementary Information

## Data Availability

The dataset is available for unlimited use under the Creative Commons License 4.0 International at Huggingface 10.57967/hf/5047. The original data sources are cited within this paper in the Methods section.

## Code availability

The code implementation to reproduce the database and is available on GitHub (https://github.com/zhu-xlab/CropClimateX). The repository includes a comprehensive README file and a tutorial to guide users through setup, usage, and potential modifications. Additionally, it includes a script to efficiently download the data from HuggingFace.
