# Supplementary material for: A large-scale, multitask, multisensory dataset for climate-aware crop monitoring in the US from 2018–2022
Source: Sci Data. 2026 Jan 20;13:72. doi: 10.1038/s41597-026-06611-x (PMC12824394; doi:10.1038/s41597-026-06611-x)
Supplement: Supplementary file 1 — Supplementary Information [file 41597_2026_6611_MOESM1_ESM.pdf]

Supplementary Information

Minicube Optimization

The fitness/goodness of an individual is determined by the weighted combination of the four variables introduced in the Minicube Sampling Strategy section: (1) the proportion of the area of the minicubes within the county to the total area of the minicubes, (2) the overlap between the minicubes with respect to the total area of minicubes, (3) the number of minicubes with respect to the number of minicubes in the baseline, and (4) the crop area contained by the minicubes with respect to the crop area contained by the county. The weights were optimized empirically using a small representative subset of counties, where the algorithm's results were qualitatively evaluated. This led to the final weights of (w1, w2, w3, w4)=(-2, +5, +1, -4). Since the Genetic Algorithm (GA) aims to minimize its objective, the lower the score, the better. This also explains the sign of the weights. We want to have (1) a high proportion of area inside compared to area outside the county, which results in a negative sign (-2), i.e., when minimizing this term, the area inside the county is maximized; (2) a low overlapping area between the county minicubes, so this term is minimized (+5); (3) a low number of minicubes, also resulting in a positive sign (+1); and (4) the largest possible crop area contained, hence a negative sign to maximize it (-4). The weights were tuned using the GA (see Algorithm 2), as the Sliding Grid Algorithm (SGA) (see Algorithm 1) does not include any overlap by design and is therefore not suitable for defining all weights. The final weights, determined through the GA, are then also used in SGA to evaluate the fitness/goodness of an individual and to compare the fitness/goodness between the algorithms, ultimately leading to the final selection of minicubes.

The weights reflect a trade-off among the different variables/objectives. While increasing one generally improves that objective's score, it can negatively impact others. For example, raising the crop proportion weight (objective 4) naturally results in more area outside, more overlap, and more minicubes because it makes it easier to cover the crop areas. Additionally, there is an interaction between the objectives when they support each other. For instance, the crop proportion objective already concentrates the minicubes within the county (since they are only in crop areas), but this does not mean the minicubes will shift inside the county near its edge. Conversely, having fewer minicubes makes it easier to avoid overlap, which supports objectives 2 and 3.

We use the two examples from the Technical Validation section, counties 29189 and 55131 (see Figure 8), to illustrate how the different objectives and their corresponding weights interact. Figure S1 shows a radial plot demonstrating how a single weight influences the minicube placement. Each axis scales the final weight by 0, 0.5, 1, 1.5, and 2, with no initial influence and increasing influence thereafter. For example, if we set the first weight to zero (w1-), which controls keeping the minicubes within the county (objective 1), it can be observed that the minicubes occupy areas outside the county, despite minimal crop presence (see county 29189 in Figure S1a). Increasing this weight causes the algorithm to try to keep the minicubes contained within the county. Conversely, a strong weight causes higher restriction, possibly leading to lost crops on the edge of the county, and overlap occurs across minicubes (w1+). Without considering the second weight (objective 2), overlapping minicubes happen (w2-), but increasing its value regulates the overlap. However, increasing it further does not improve the overlap (w2+). The same applies to the third weight, which limits the number of minicubes (objective 3). A low weight (w3-) yields more minicubes, while increasing it further shows little effect (w3+). Finally, without the fourth weight (w4-), crop regions are not selected. While increasing the weight results in more crop coverage (objective 4), this also raises the number of minicubes, which only cover small parts of the crops, leading to a slight increase in crop coverage (w4+). The same conclusions can be drawn for county 55131 in Figure S1b. In contrast, even without the crop (w4-), crop regions are still selected, as the crops are evenly distributed within the county, and the other weights guide the algorithm inside the county. Interestingly, in this case, only one minicube is used, possibly because the allocation (w1) can be achieved with fewer minicubes, so the only way to optimize further is by reducing the number of minicubes (w3).

We now illustrate the pairwise interactions of the weights in Figure S2. The first row of subplots displays the combination with the first weight, while the second row of subplots shows the remaining interactions for weights 2 to 4. Given the final weights, we again scale them by 0.5 and 2 for all possible combinations of these two weights. These combinations are presented around the final solution in the center to demonstrate their interactions. For instance, the first subplot, located in the top left for both counties, shows the interaction between the county allocation and the overlap between the minicubes (w1 vs w2). Decreasing the weight of these objectives leads to higher crop proportions, but also to more minicubes and overlap. Conversely, increasing both weights results in a smaller crop proportion and fewer minicubes. Enlarging one and reducing the other, or vice versa, leads to more overlap or a smaller allocation of the county's area. The next subplot (w1 vs w3) does not change significantly for county 29189. However, for county 55131, relaxing these weights results in more overlap and area outside the county. Other combinations also allow more area outside or reduce the number of minicubes. As mentioned in the County and Minicube Sampling section in the Technical Validation, the algorithm has difficulties placing the minicubes when the crop is evenly distributed. Therefore, there are also better solutions (which can be achieved by chance, as seen on the top right, where w1 is decreased and w3 is increased). The top right subplot indicates that increasing the weight of the objective 1 captures more crops, but also increases the number of minicubes. Only increasing the weight of the allocation can counteract this effect.

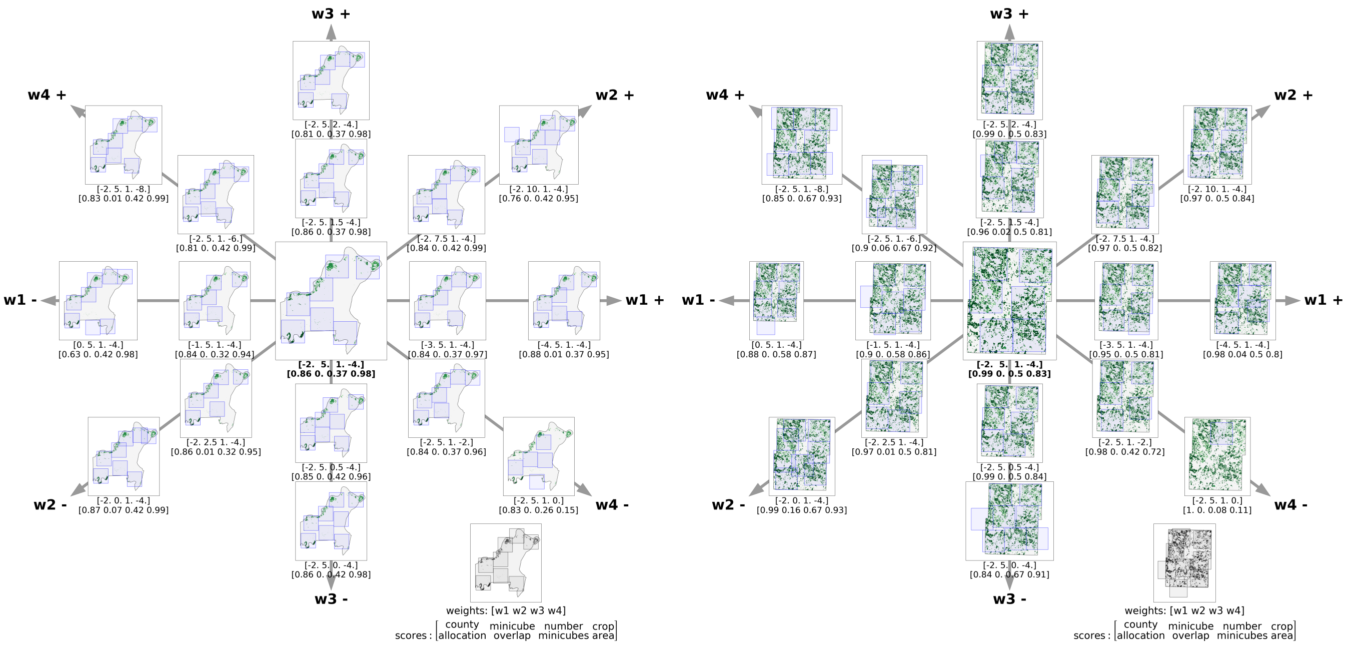

(a) County 29189. **(b)** County 55131.

Figure S1. Visualization of the variable/objective weights interactions to the final solution using the GA for counties 29189 and 55131. The radial plots show the increase/decrease of a single weight in one direction, starting from the final weights visualized in the middle. Each final weight is multiplied by 0, 0.5, 1.5, and 2 to provide the four variations along an axis, showing the influence of that parameter at different levels. The accumulated number of crops is in green, the sampled minicubes are in blue, and the county outline is in black. Below each plot are the four weights and the scores achieved by each objective.

However, placing more minicubes within the county leads to overlapping minicubes. The bottom left visualization (w2 vs w3) reveals that heavily weighting the overlap between minicubes results in more area outside the county. Moreover, relaxing both terms allows placing more minicubes within a county, allowing overlapping structures. This could be a favorable strategy for county 55131, as it achieves a higher crop score. However, for county 29189, where the crop area is more clustered, this simply creates more overlap without affecting the crops. In the next subplot (w2 vs w4), increasing or decreasing each weight does not change the result favorably because it creates more minicubes, which may extend outside the county or result in increased overlap between the minicubes. Finally, the subplot in the bottom right (w3 vs w4) demonstrates that increasing the weighting of the crop proportion cannot be offset by raising the weight for the number of minicubes, ultimately resulting in greater overlap and additional area outside of the county.

The parameters of the algorithms were evaluated in the same way as the weights. A description of these parameters and their actual values is provided in Tables S1 and S2. Some parameters are scaled linearly with the size of the county, as the number of possible minicube placements (without overlaps) scales linearly with the area to cover. For example, the number of training generations, the population size, and the noise decay of the GA are scaled linearly with the size of the county.

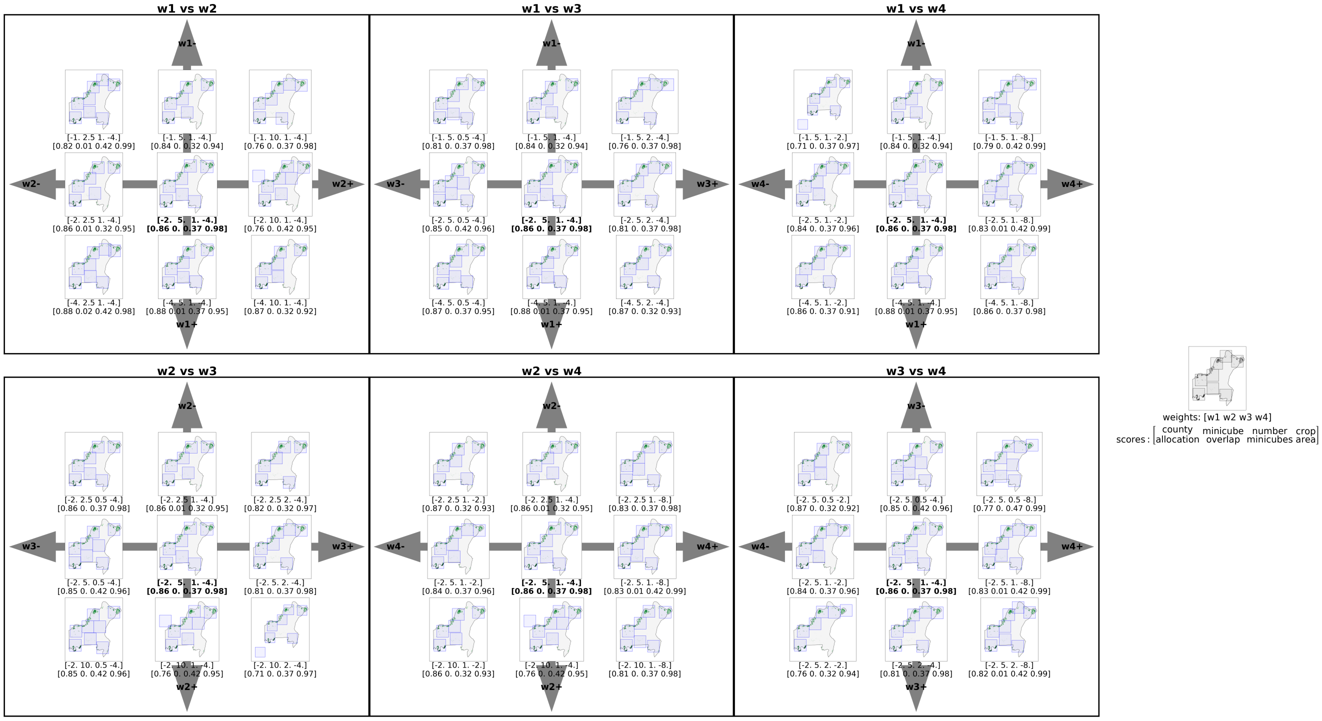

(a) County 29189.

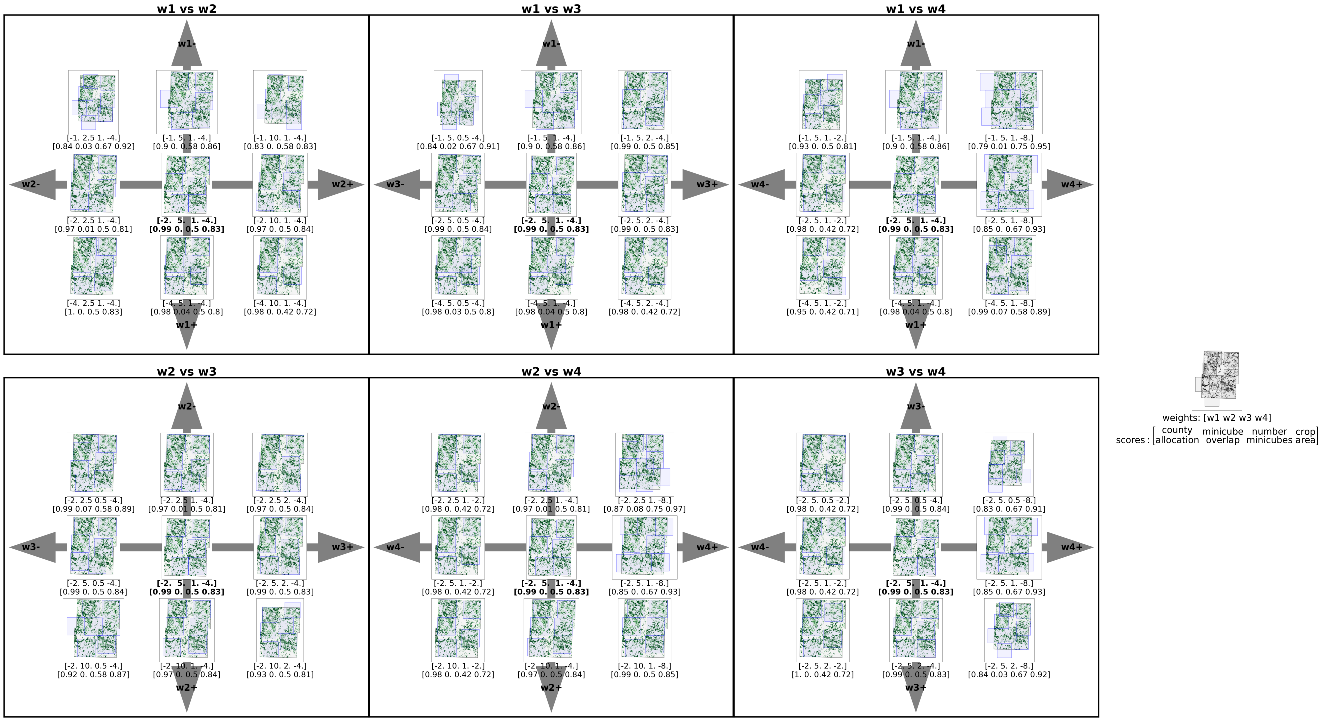

(b) County 55131.

Figure S2. Visualization of the variable/objective weight interactions to the final solution using the GA for counties 29189 and 55131. The final weights are pairwise varied by multiplying by 0.5 and 2 to provide each combination of the two weights in a separate subplot. The accumulated number of crops is in green, the sampled minicubes are in blue, and the county outline is in black. Below each plot are the four weights and the scores achieved by each objective.

Table S1. Parameters used for the SGA.

Name	Description	Parameter
pixel shift	The number of pixels the grid shifts by. This parameter scales linearly with the size of the county to manage the algorithm's runtime. Because the crops in the counties are mostly spread evenly or clustered (see Section Technical Validation - County and Minicube Sampling), this does not greatly affect their performance.	1–6
$thres_{crop}$	The percentage of crop area that at least one minicube must contain in the grid. The parameter is scaled based on the number of minicubes in the grid. If there were only one minicube, it would need to hold 50% of the crop.	-
$thres_{crop2}$	The percentage of crop a minicube must have to ignore the allocation score. This prevents minicubes from being removed when crops are near the county boundary and cannot be placed elsewhere. This value adjusts dynamically based on the number of minicubes in the grid.	-
$thres_{area}$	The proportion of the area of the minicube that must be within the county.	0.4

Table S2. Parameters used for the GA.

Name	Description	Parameter
algorithm	The genetic algorithm used to train the model. We decided to follow a simple yet effective algorithm, known as `eaSimple` in DEAP. It applies the basic operations of a genetic algorithm. More advanced algorithms automatically adapt the search distribution over the parameters (Covariance Matrix Adaptation), but they require higher computational costs.	eaSimple
n_{pop} and n_{gen}	The population size and the number of generations the algorithm runs. These parameters determine the primary factors that affect its runtime. Since the number of possible minicube placements without overlap scales linearly with the size of the area to be covered, and computation time increases significantly with these parameters, we adjusted these numbers accordingly. Further, higher population and generation sizes did not provide any additional benefits.	300-3500 and 300-1200
cxpb	The probability of applying cross-over/mate to generate new offspring.	0.1
mutpb	The probability of mutating (adding noise or removing/adding minicubes) to generate new offspring.	0.2
noisepb	The probability of adding noise or removing/adding a minicube to generate new offspring.	0.9
noise percentage	The probability of adding noise to a minicube from an individual after the individual was selected for noise injection.	0.5
noise_range	Number of pixels the noise introduces.	200
noise decay	The decay of noise in each epoch follows an exponentially decaying schedule. It scales proportionally with the size of the county. Noise decay is inversely related to the size of the county: the larger the county, the longer the high noise level persists, since they are trained for longer periods.	55-210
tournsize	The size of the offspring individuals compared at each roulette step to create the next generation. It scales linearly with the size of the county because the population size also increases proportionally to the county's size.	3-12
early stop	Terminating the algorithm after a certain number of generations when there is no improvement in the best individuals. It is assumed that the algorithm has reached a local minimum, and to reduce computation time, the optimization is stopped.	75
